# Intrathoracic Vagal Schwannoma Mimicking Metastatic Lymphadenopathy with a Positional Shift

**DOI:** 10.70352/scrj.cr.24-0190

**Published:** 2025-11-15

**Authors:** Rina Takahashi, Tomonari Oki, Shuhei Iizuka, Yoshiro Otsuki, Toru Nakamura

**Affiliations:** 1 Departments of General Thoracic Surgery, Seirei Hamamatsu General Hospital, Hamamatsu, Shizuoka, Japan; 2 Departments of Pathology, Seirei Hamamatsu General Hospital, Hamamatsu, Shizuoka, Japan

**Keywords:** neurilemmoma, vagus nerve, lymphadenopathy, ^18^F-fluorodeoxyglucose, paraneoplastic syndromes

## Abstract

**INTRODUCTION:**

Schwannomas are benign tumors originating from peripheral nerve sheaths and are most commonly found in the head, neck, and extremities. Intrathoracic schwannomas, particularly those arising from the vagus nerve, are relatively rare. Despite their benign nature, schwannomas often exhibit fluorodeoxyglucose (FDG) uptake on PET scans, potentially leading to diagnostic confusion. This report presents a case initially suspected to be a metastatic lymph node but later diagnosed as a schwannoma from the intrathoracic vagus nerve, highlighting the diagnostic challenges associated with thoracic nodal lesions.

**CASE PRESENTATION:**

A 36-year-old woman with a history of cryptogenic optic neuritis presented with a posterior mediastinal nodule discovered on chest CT. FDG-PET/CT revealed uptake in the nodule, initially raising suspicion of metastatic lymphadenopathy. A repeat CT scan showed that the nodule had shifted medially, suggesting a pedunculated lesion rather than lymphadenopathy. Thoracoscopic findings revealed a 2-cm nodule arising from the vagus nerve and exhibited mobility due to its pedunculated growth. The tumor was enucleated, preserving the main nerve trunk. Histopathological examination confirmed the diagnosis of schwannoma.

**CONCLUSIONS:**

Schwannomas originating from the thoracic vagus nerve can exhibit pedunculated growth and positional migration, posing diagnostic challenges. Mobile mediastinal nodules should prompt consideration of vagal nerve schwannomas in the differential diagnosis. Recognizing that benign schwannomas can accumulate FDG on PET scans is crucial to avoid misdiagnosis as malignancy. This case underscores the importance of comprehensive imaging analysis and clinical correlation in the evaluation of mediastinal masses, potentially alleviating unnecessary psychological burden on patients due to the suspicion of malignancy.

## Abbreviations

^18^F-FDG
: ^18^F-fluorodeoxyglucose
GLUT
: glucose transporter
ICSs
: intercostal spaces
OD
: oculus dexter
OS
: oculus sinister
SUVmax
: maximum standardized uptake value

## INTRODUCTION

Schwannomas are benign tumors originating from the peripheral nerve sheath and are most commonly found in the head and neck region or extremities.^1)^ However, schwannomas arising in the thoracic cavity, particularly from the vagus nerve, are relatively rare.^2–4)^ Despite their benign nature, schwannomas are often reported to exhibit an ^18^F-FDG uptake on PET scans, which can result in diagnostic confusion. We herein report a case that was initially suspected to be a metastatic lymph node tumor but manifested a positional shift and was eventually diagnosed as a schwannoma from the intrathoracic vagus nerve. This case underscores the diagnostic pitfalls associated with thoracic nodal lesions and highlights the significance of imaging findings and the clinical course.

## CASE PRESENTATION

A 36-year-old woman presented with a posterior mediastinal nodule found on chest CT. Three months prior, she developed cryptogenic optic neuritis, presenting with bilateral ocular pain and visual impairment, with a best-corrected visual acuity of 20/200 in the right eye (OD) and 20/1000 in the left eye (OS). Visual acuity of 20/200 means that an individual must be at 20 feet to see what a person with normal vision can see at 200 feet, and OD stands for oculus dexter, meaning “right eye” in Latin.^5)^ Following 6 days of intravenous methylprednisolone at a dose of 1 g per day, her visual acuity improved to 20/22 (OD) and 20/40 (OS). Her medical history also included chronic sinusitis and recurrent habitual miscarriages. She was currently smoking, with an 8 pack-year smoking history, and never drank any alcohol. She was not on any medications.

**Fig. 1:**
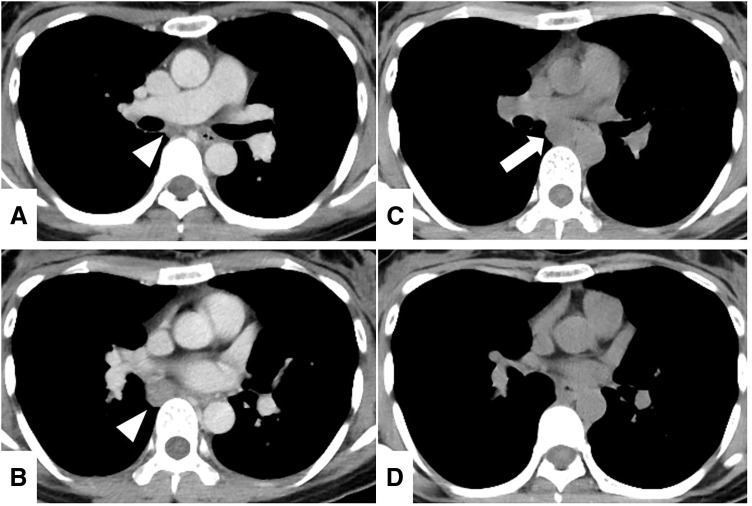
(**A**, **B**) Chest CT at the initial presentation revealed a well-circumscribed nodule along the right bronchus intermedius (arrowheads). (**C**, **D**) Chest CT 1 month later revealed that the nodule had shifted toward the midline in a cephalad direction (arrow).

**Fig. 2:**
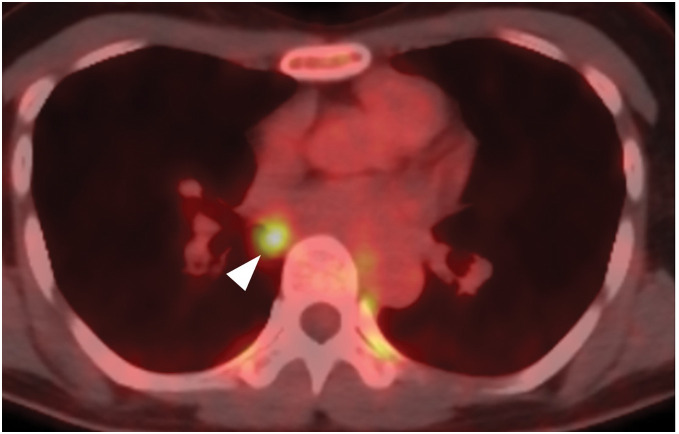
FDG-PET revealed high uptake in the nodule with an SUVmax of 5.67 (arrowhead). FDG, fluorodeoxyglucose; SUVmax, maximum standardized uptake value

A chest CT during a further evaluation of the cryptogenic optic neuritis revealed a well-circumscribed nodule along the right bronchus intermedius without any other hilar or mediastinal lymphadenopathy (**Fig. 1A** and **1B**). An ^18^F-FDG PET/CT conducted for paraneoplastic screening demonstrated an FDG uptake only in the nodule with an SUVmax of 5.67 (**Fig. 2**). The nodule was considered to be a hypermetabolic lymphadenopathy, such as a metastasis, but there were no other lesions suspicious of a primary malignancy. We suspected the nodule to be a metastatic mediastinal lymphadenopathy from an unknown primary tumor, that is, stage 4 disease, and planned a biopsy via endobronchial ultrasound.

A repeat contrast-enhanced chest CT scan performed to assess the perilesional blood supply showed that the nodule had shifted medially as compared to the initial CT (**Fig. 1C** and **1D**), and its mobility raised suspicion of a pedunculated lesion, such as a solitary fibrous tumor, rather than a lymphadenopathy.^6,7)^ We altered the plan and decided to perform a thoracoscopic curative resection instead of a surgical biopsy. Transesophageal or bronchial needle aspiration was omitted due to the lesion’s mobility, as the potential benefits of a puncture were considered minimal given the concerns regarding safety and diagnostic uncertainty.

**Fig. 3:**
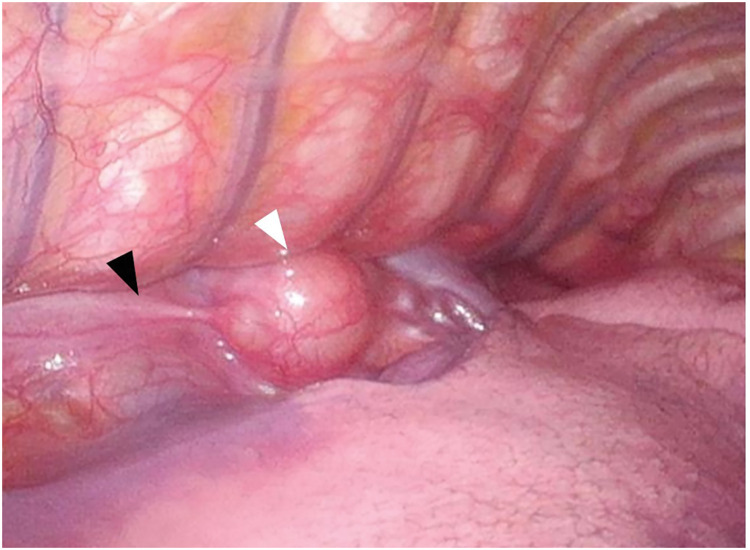
Thoracoscopic findings revealed a nodule (white arrowhead) arising from the vagus nerve (black arrowhead) and exhibited mobility due to its pedunculated growth.

**Fig. 4:**
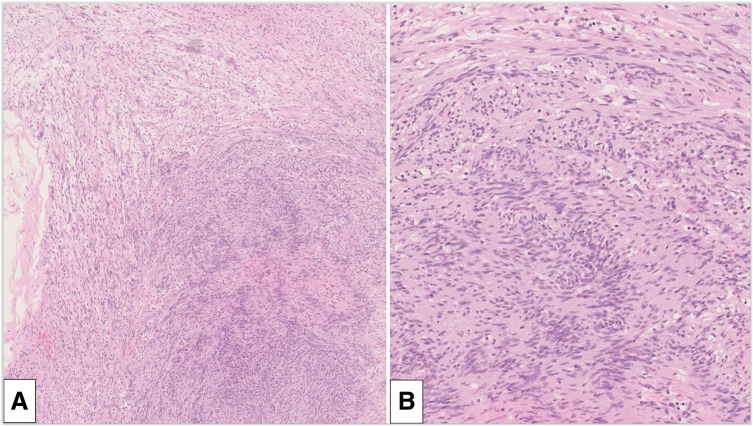
(**A**) Pathological findings revealed coarse and fine multiple spindle cells with a fenestrated array pattern. Hypocellularity with a predominantly loose myxoid component was combined (Antoni B) (×40). (**B**) The spindle cell proliferation was characterized by an alternating highly ordered cellular Antoni A component.

With the patient in the prone position, two 5-mm trocars were inserted in the 5th and 9th ICSs, and a 12-mm trocar was placed in the 7th ICS along the posterior axillary line. Carbon dioxide was insufflated at 8 mmHg and revealed a well-circumscribed nodule approximately 2 cm in size, located along the esophagus (**Fig. 3**). The nodule was found to arise from the vagus nerve and exhibited mobility due to its pedunculated growth. It was enucleated at the subpleural level, preserving the main nerve trunk. Histopathological examination revealed coarse and fine multiple spindle cells with a fenestrated array pattern, consistent with a schwannoma (**Fig. 4**).

The postoperative course was uneventful, and the patient was discharged on the 1st POD. One year after surgery, she remains free of visual impairment without the need for any additional medication.

## DISCUSSION

Schwannomas are one of the most common mediastinal neoplasms in adults.^8)^ They typically present as well-circumscribed, smooth-margined nodules with a homogeneous internal structure, most frequently located in the posterior mediastinum. In the present case, we initially suspected the nodule to be a metastatic lymph node due to its subcarinal location and FDG accumulation during the initial workup. However, the 2nd CT revealed a positional shift, suggesting a different type of stalked lesion rather than a mediastinal lymph node, which is typically fixed beneath the parietal pleura. Although the well-defined nodule with smooth margins in the mediastinum could have raised a schwannoma as a differential diagnosis, the positional shifting and FDG uptake led to diagnostic confusion. The most common origins of intrathoracic schwannomas are the sympathetic chain and intercostal nerves, which are firmly anchored to the thoracic vertebrae or ribs.^2,8–11)^ In contrast, the thoracic vagus nerve in the middle and lower mediastinum forms a loose plexus around the esophagus as it travels along its course. These anatomical findings suggest that tumors arising from the thoracic vagus nerve could possess an exceptional potential for positional migration. Schwannomas arising in other locations may also exhibit mobility; however, this is less common.^12,13)^ In addition to the rarity of intrathoracic vagal schwannomas, the shifting nature of the lesion posed a diagnostic challenge.

In the present case, PET imaging was performed because the ocular symptoms were suspected to be a manifestation of paraneoplastic syndrome, a rare disorder triggered by an altered immune system response to a neoplasm. This condition can cause a wide range of neurological symptoms that could affect various parts of the nervous system, including the ocular syndromes, as shown in the present case.^14,15)^ The relationship between the ocular symptoms and the schwannoma remained unclear because the patient’s symptoms had completely resolved with methylprednisolone administration before the surgery. The suspicion of paraneoplastic syndrome, misdiagnosis of the nodule as a lymph node, and FDG accumulation led to a heightened concern for a malignancy.

It is well known that benign schwannomas can show a high FDG uptake with an unknown etiology.^16–18)^ The hypotheses include a higher cellularity, increased local metabolic activity, and an inflammatory response around the tumor; however, these are not associated with the tumor size, Ki-67 index, or GLUT expression levels.^17,19)^ This false-positive finding could lead to a misdiagnosis as metastatic disease, even in cases where the disease is at a curative stage.^18,20,21)^ In the present case, a malignancy was suspected primarily due to the FDG accumulation, which placed a psychological burden on the patient, raising unnecessary concerns that the condition might be incurable.

Had a “benign schwannoma” been included in the differential diagnosis during the initial workup, it might have been possible to avoid the psychological burden associated with the suspicion of an incurable disease for the patient.

## CONCLUSIONS

Schwannomas originating from the thoracic vagus nerve can develop a pedunculated growth and exhibit a positional migration. A mobile mediastinal nodule should raise consideration of a vagal nerve schwannoma in the differential diagnosis. Recognizing that FDG can accumulate in benign schwannomas allows for a diagnostic workup that avoids undue fixation on a malignancy, potentially alleviating any unnecessary psychological burden on the patient.

## SUPPLEMENTARY MATERIALS

Supplementary Video Intraoperatively, the nodule was found to be loosely attached and demonstrated mobility.

## References

[1] Das Gupta TK, Brasfield RD, Strong EW, et al. Benign solitary Schwannomas (neurilemomas). Cancer 1969; 24: 355–66. 5796779 10.1002/1097-0142(196908)24:2<355::aid-cncr2820240218>3.0.co;2-2

[2] Zhu W, Chen D. Vagus nerve schwannoma in the right upper mediastinum. Thorac Cancer 2017; 8: 698–702. 28805352 10.1111/1759-7714.12485 PMC5668479

[3] Dabir RR, Piccione W Jr., Kittle CF. Intrathoracic tumors of the vagus nerve. Ann Thorac Surg 1990; 50: 494–7. 2205165 10.1016/0003-4975(90)90509-5

[4] Heitmiller RF, Labs JD, Lipsett PA. Vagal schwannoma. Ann Thorac Surg 1990; 50: 811–3. 2241349 10.1016/0003-4975(90)90692-y

[5] Azzam D, Ronquillo Y. Snellen Chart. Treasure Island (FL): StatPearls Publishing; 2025. 32644387

[6] Watanabe K, Takabe Y, Iizuka S, et al. Solitary fibrous tumor of the pleura mimicking a soft tissue sarcoma of the chest wall. Int J Surg Case Rep 2022; 91: 106746. 35026682 10.1016/j.ijscr.2021.106746 PMC8760409

[7] Berne AS, Heitzman ER. The roentgenologic signs of pedunculated pleural tumors. Am J Roentgenol Radium Ther Nucl Med 1962; 87: 892–5. 13868118

[8] Davis RD Jr., Oldham HN Jr., Sabiston DC Jr. Primary cysts and neoplasms of the mediastinum: recent changes in clinical presentation, methods of diagnosis, management, and results. Ann Thorac Surg 1987; 44: 229–37. 2820323 10.1016/s0003-4975(10)62059-0

[9] Fierro N, D’Ermo G, Di Cola G, et al. Posterior mediastinal schwannoma. Asian Cardiovasc Thorac Ann 2003; 11: 72–3. 12692029 10.1177/021849230301100118

[10] Oosterwijk WM, Swierenga J. Neurogenic tumours with an intrathoracic localization. Thorax 1968; 23: 374–84. 5664698 10.1136/thx.23.4.374 PMC471805

[11] Duwe BV, Sterman DH, Musani AI. Tumors of the mediastinum. Chest 2005; 128: 2893–909. 16236967 10.1378/chest.128.4.2893

[12] Savu C, Grigorie V, Melinte A, et al. Giant intrathoracic Schwannoma: a case report. In Vivo 2020; 34: 3527–32. 33144463 10.21873/invivo.12194 PMC7811636

[13] Terada Y, Toda H, Yokote A, et al. A mobile schwannoma of the cervical spinal cord: case report and review of the literature. Neurosurgery 2016; 78: E156–9. 26287552 10.1227/NEU.0000000000000975

[14] Pelosof LC, Gerber DE. Paraneoplastic syndromes: an approach to diagnosis and treatment. Mayo Clin Proc 2010; 85: 838–54. 20810794 10.4065/mcp.2010.0099 PMC2931619

[15] Sarkar P, Mehtani A, Gandhi HC, et al. Paraneoplastic ocular syndrome: a pandora’s box of underlying malignancies. Eye (Lond) 2022; 36: 1355–67. 34345027 10.1038/s41433-021-01676-x PMC9232643

[16] Wang SY, Liu JH, Yao S, et al. PET/CT and contrast-enhanced CT imaging findings in benign solitary schwannomas. Eur J Radiol 2021; 141: 109820. 34139574 10.1016/j.ejrad.2021.109820

[17] Miyake KK, Nakamoto Y, Kataoka TR, et al. Clinical, morphologic, and pathologic features associated with increased FDG uptake in schwannoma. AJR Am J Roentgenol 2016; 207: 1288–96. 27657364 10.2214/AJR.15.14964

[18] Boré P, Descourt R, Ollivier L, et al. False positive 18F-FDG positron emission tomography findings in schwannoma—a caution for reporting physicians. Front Med (Lausanne) 2018; 5: 275. 30349818 10.3389/fmed.2018.00275 PMC6186987

[19] Beaulieu S, Rubin B, Djang D, et al. Positron emission tomography of schwannomas: emphasizing its potential in preoperative planning. AJR Am J Roentgenol 2004; 182: 971–4. 15039173 10.2214/ajr.182.4.1820971

[20] Kang S. Benign schwannoma mimicking metastatic lesion on F-18 FDG PET/CT in differentiated thyroid cancer. Nucl Med Mol Imaging 2013; 47: 138–40. 24900096 10.1007/s13139-013-0194-8 PMC4041980

[21] Fujiuchi N, Saeki T, Takeuchi H, et al. A false positive for metastatic lymph nodes in the axillary region of a breast cancer patient following mastectomy. Breast Cancer 2011; 18: 141–4. 19554397 10.1007/s12282-009-0125-9

